# Supplementary material for: Articulation and growth of skeletal elements in balanid barnacles (Balanidae, Balanomorpha, Cirripedia)
Source: R Soc Open Sci. 2019 Sep 4;6(9):190458. doi: 10.1098/rsos.190458 (PMC6774972; doi:10.1098/rsos.190458)
Supplement: Supplementary figures S1 to S5 [file rsos190458supp1.pdf]

Articulation and growth of skeletal elements in balanid barnacles (Balanidae, Balanomorpha, Cirripedia)

Antonio G. Checa[1,2], Carmen Salas[3], Alejandro B. Rodríguez-Navarro[4],Christian Grenier[1], Nelson A. Lagos[5]

[1]Departamento de Estratigrafía y Paleontología, Universidad de Granada, 18071 Granada, Spain, [2]Instituto Andaluz de Ciencias de la Tierra, CSIC-Universidad de Granada, 18100 Armilla, Spain, [3]Departamento de Biología Animal, Facultad de Ciencias, Universidad de Málaga, 29071 Málaga, Spain, [4]Departamento de Mineralogía y Petrología, Universidad de Granada, 18071 Granada, Spain, [5]Centro de Investigación e Innovación para el Cambio Climático, Universidad Santo Tomás, Santiago, Chile

Supplementary figures S1 to S5 and caption to Supplementary video S1

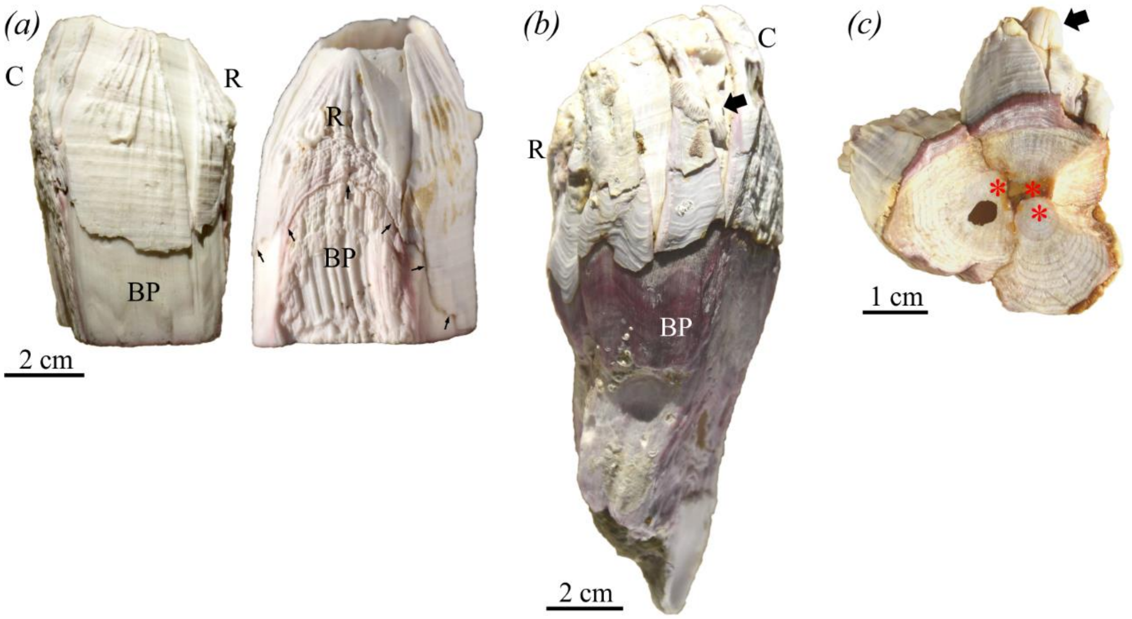

Supplementary figure S1. Growth plasticity in *A. psittacus*. *(a)* Right and anterior views of a specimen showing changes in extension of the wall plates relative to the basal plate, due to impingement from other neighbours. The thin arrows in the right image indicate the boundary of the basal plate. *(b)* Specimen with a particularly large vertical development of the basal plate. *(c)* Basal view of a group of three specimens. The asterisks indicate the apex of the basal plates. The plates became displaced preferentially away from the contact with the neighbours during growth. The thick arrows in *(b)* and *(c)* indicate the positions of small specimens which grew at the boundaries between plates, thus preventing further growth at those contacts. BP: basal plate; C: carina; R: rostrum.

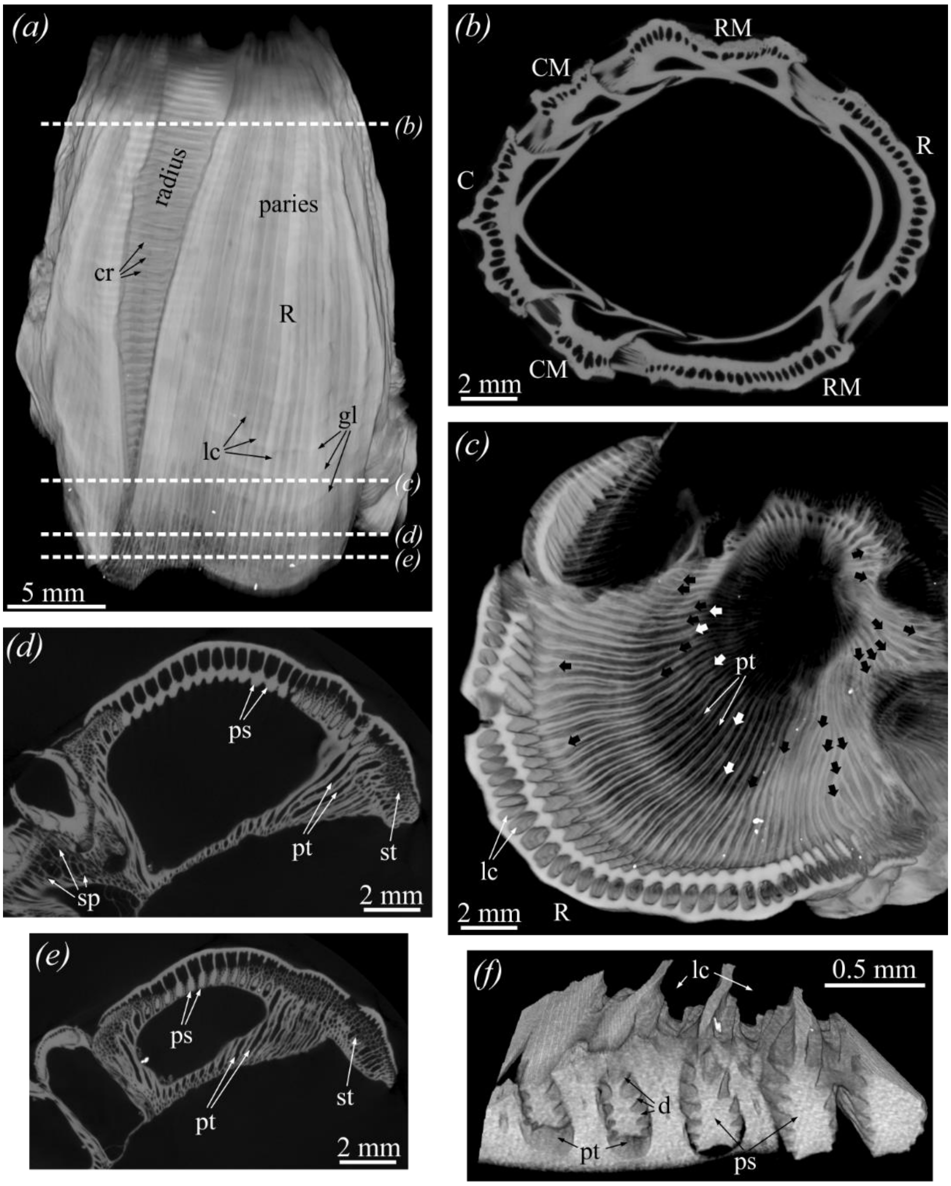

Supplementary figure S2. Micro-CT analysis of *A. psittacus*. *(a)* External view of a specimen. The semi-transparency of the image allows us to discern the crenulations of the radii and the longitudinal canals of the paries. *(b)*-*(e)* Horizontal sections at different levels of the specimen shown in *(a)* (indicated). *(b)* Section showing the different structures close to the ventral side. Compare to figure 1*b*. *(c)* Section close to the basal plate. Note the radial arrangement of primary tubes of the basal plate. The arrows point to the approximate positions where primary tubes split. *(d)*, *(e)* Sections at the interface between the wall and basal plates. Depending on the level, the development of primary vs subsidiary tubes change, with these being predominant closer to the substrate (panel *(e)*). The sectioned subsidiary tubes provide a vesicular framework. Note septations within subsidiary tubes. *(f)* Detail of the interlocking between the primary septa of the lateral plate and the openings of the primary tubes of the basal plate. There are permanent gaps between the denticles of the primary septa and the walls of the primary tubes. C: carina; CM: carinomarginal; cr: crenulations; d: denticles; gl: growth lines; lc: longitudinal canals; ps: primary septa; pt: primary tubes; R: rostrum; RM: rostromarginal; sp: septa within subsidiary tubes; st: subsidiary tubes.

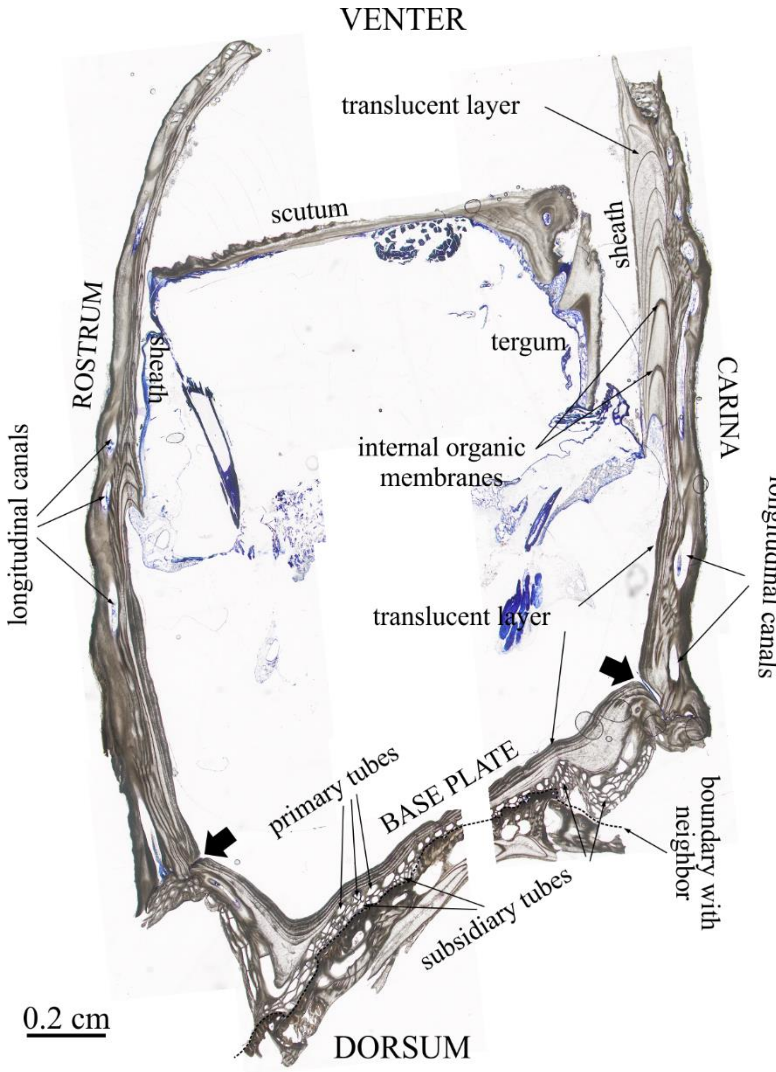

Supplementary figure S3. Dorsoventral thin section through the carina and rostrum of the shell of a methacrylate-embedded specimen of *A. psittacus*, showing the main morphological elements. The thick arrows indicate the boundaries between plates. The internal translucent layer spreads onto the interior of the base and the wall plates, and forms the sheath. The internal organic membranes conform to the outline of the sheath. Remains of cellular material (stained in blue) can be observed within the longitudinal canals and within the boundaries between the wall plates and the basal plate.

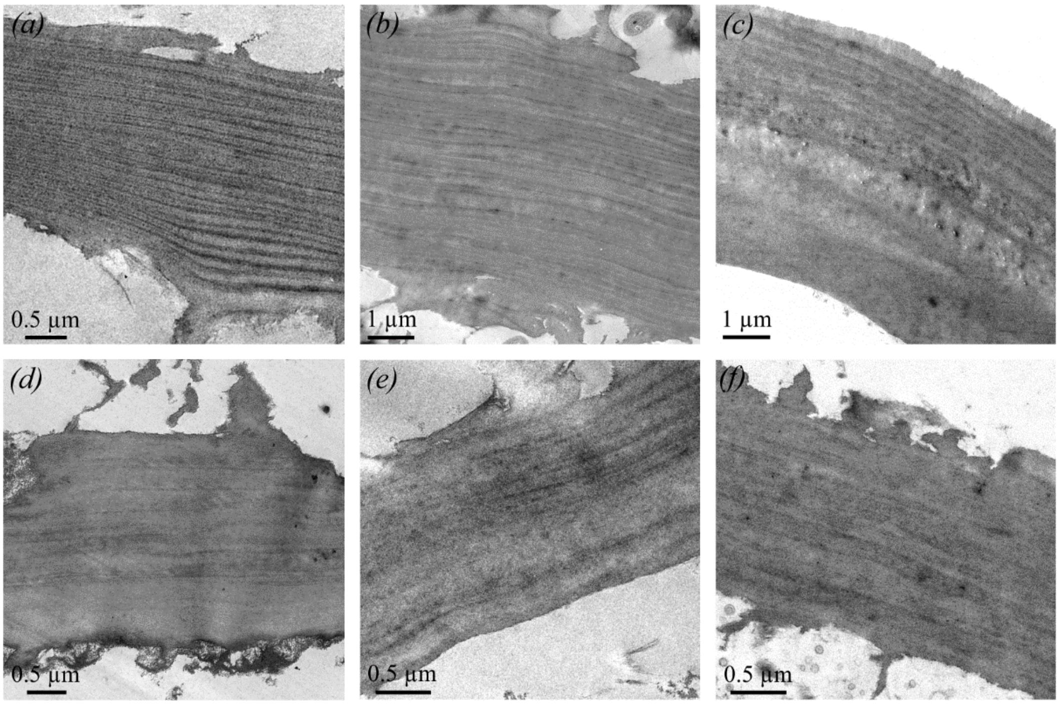

Supplementary figure S4. TEM of membranes of *A. psittacus* extracted from different areas of the plates and plate margins. *(a)* Cuticle. *(b)* Membrane of the interior of the wall plate. *(c)* Membrane covering the rostral margin of the paries. *(d)* Membrane between the rostral margin of the ala and the carinal margin of the sheath. *(e)* Membrane covering the periphery of the base plate. *(f)* Membrane lining the primary tubes of the plate base.

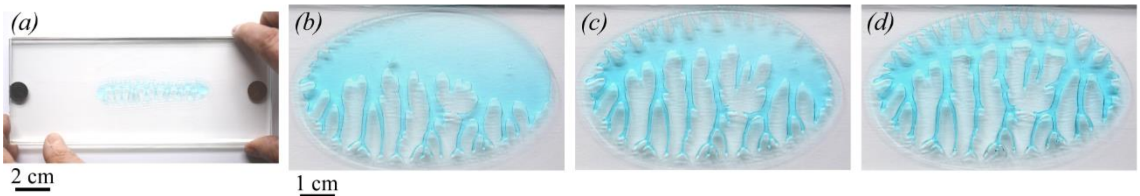

Supplementary figure S5. Formation of elongated viscous fingers using the lifting procedure. *(a)* Experimental setting. Two methacrylate plates, between which a stretched mass of gel (light blue colour) has been placed, are separated by two thin metal disks. The upper plate is then pressed against the lower plate, such that the gel in between spreads. When the pressure is released, the two plates separate gradually to the distance imposed by the thickness of the metal disks. This induces a drop in the internal pressure of the gel, and consequently, the surrounding air is injected into the gel. *(b)*-*(d)* Progressive formation of a viscous fingering pattern. The injection of air into the gel provokes an instability, in such a way that the interface between the two fluids becomes increasingly dendritic.

Supplementary video S1. Journey through the interior of a specimen of *A. psittacus*. Same specimen as in figure S2*a*-*e*.
